# Centre of the Cell: Science Comes to Life

**DOI:** 10.1371/journal.pbio.1002240

**Published:** 2015-09-04

**Authors:** Frances Balkwill, Katie Chambers

**Affiliations:** 1 Centre of the Cell, Queen Mary University of London, London, United Kingdom; 2 Centre for Cancer and Inflammation, Barts Cancer Institute, Queen Mary University of London, London, United Kingdom

## Abstract

Centre of the Cell is a unique biomedical science education centre, a widening participation and outreach project in London’s East End. This article describes Centre of the Cell’s first five years of operation, the evolution of the project in response to audience demand, and the impact of siting a major public engagement project within a research laboratory.

## Abstract 2

This Community Page describes "Centre of the Cell"—an innovative and successful public engagement project situated inside a research laboratory in London's East End—and its impact on research staff and local audiences.

Centre of the Cell is a unique cell-shaped science centre suspended above a real biomedical research laboratory in the heart of London’s East End. It is one of the few, perhaps the only, science education centres in the world to be situated inside a research lab—in the Blizard Institute at the Whitechapel medical and dental campus of Queen Mary University of London (QMUL). Since its opening in September 2009, over 100,000 people have participated in Centre of the Cell activities (Fig 1) with approximately one million visits to the interactive website www.centreofthecell.org. With visitor numbers and activities increasing year on year, we believe this is an example of an innovative and successful public engagement project that may serve as an example for similar initiatives in biomedical research institutes.

**Fig 1:**
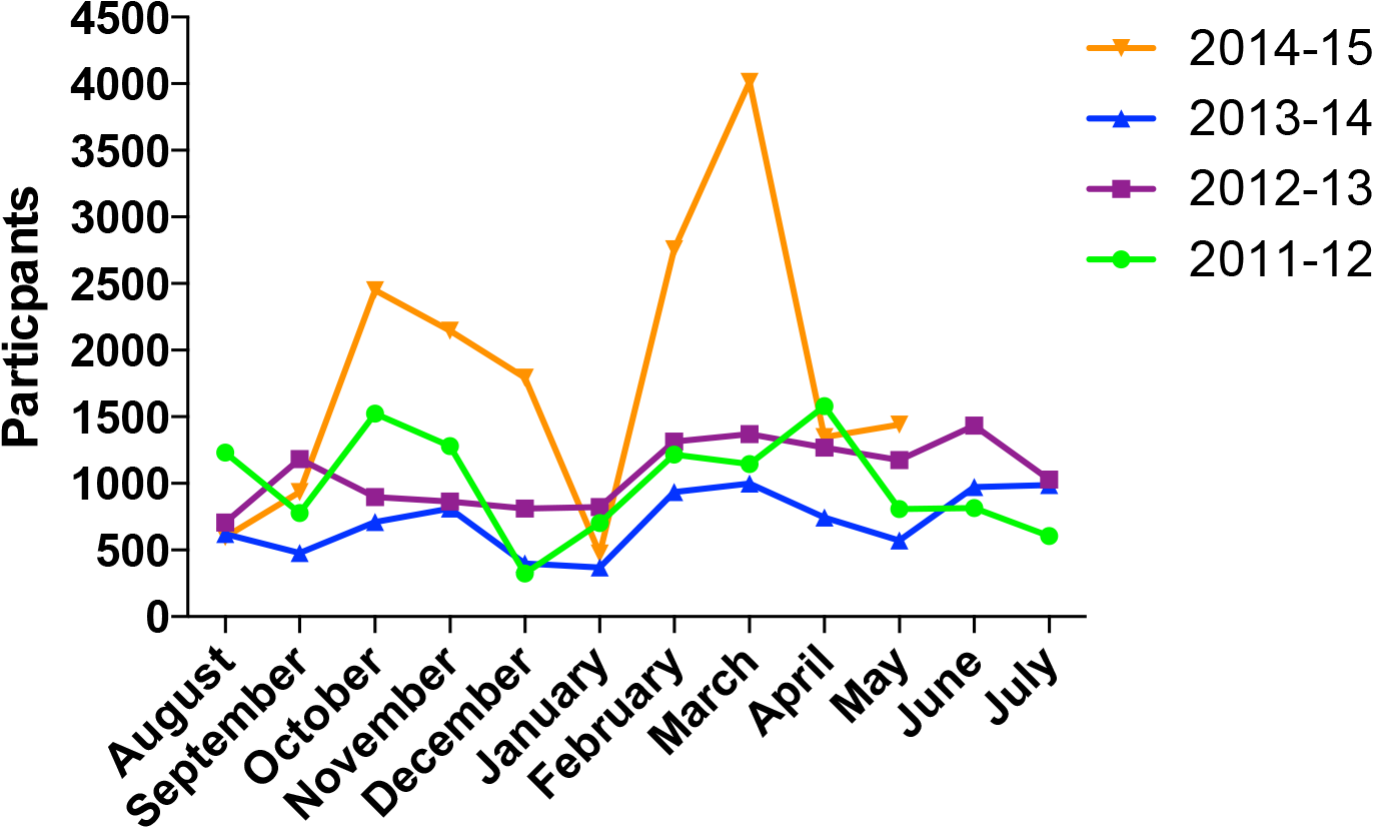
Visitor statistics for Centre of the Cell years 3–6 (2011–2015). These monthly statistics include all visitors on site as well as visitors to our workshops and shows in schools and other locations. Year 1 and 2 figures (not shown here) were 15,387 and 19,585, respectively. These data show a consistent pattern from month to month. 2013–2014 numbers were lower, due to planned maintenance shutdown of the Pod and introduction of charging for Pod shows that led to a dip in visitor numbers that largely recovered during 2014.

## Origins of Centre of the Cell

The motivation for Centre of the Cell was the local population of the East End, an area of social deprivation and poor health, with a large immigrant population. There was a need to inspire and motivate the local school children into higher education and to site a science centre in an area that would not traditionally be anticipated to draw a large audience. Prior to opening in 2009, there was an extensive front-end evaluation of the project plan with the local audience, as described in more detail below.

**Fig 2:**
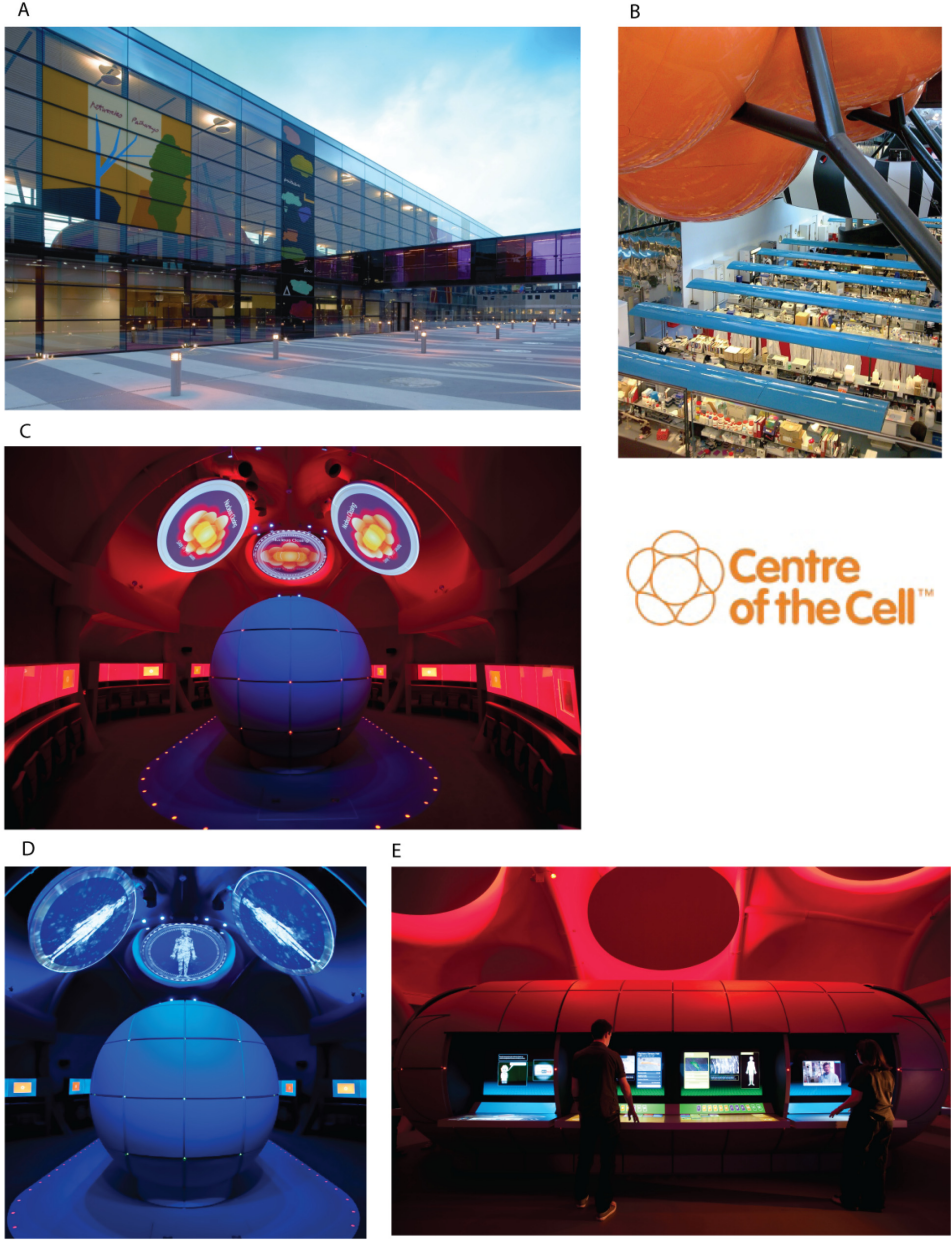
Location of Centre of the Cell within the Blizard Research Institute. **A)** Visitors access the orange Centre of the Cell Pod (arrow) via a multicoloured glass bridge. **B)** The Pod is suspended above the research laboratories, allowing visitors to see scientists at work. Images 2A and 2B courtesy of Centre of the Cell. **C)** and **D)** The first scenes of the Pod show—an introduction to cell biology is displayed on overhead circular screens while the central nucleus remains closed. **E)** The nucleus has now opened, allowing visitors to play interactive games related to cell biology. Images 2C, 2D, and 2E courtesy of Nick Wood for Land Design Studio.

Ten years ago, the Whitechapel science campus of QMUL was rather desolate and disorganised; the development of the Blizard Institute, in which Centre of the Cell is housed, was intended to reverse many years of lack of investment. An innovative design from architect Will Alsop resulted in a vast subterranean laboratory floor housing 400 scientists, covered by a rectangular glass box housing offices and “Pod” meeting rooms (see Fig 2). Thanks to the foresight of microbiologist Professor Mike Curtis (who subsequently became the first director of the Blizard Institute and has been a major contributor to Centre of the Cell’s success), a space for public engagement was part of the original architectural brief. Hence, one of the Pods in the glass box took shape as a bright orange cell-shaped structure suspended above the lab benches.

## Centre of the Cell’s Aims

Centre of the Cell has five main aims: to inspire the next generation of scientists and healthcare professionals; stimulate dialogue, interest, and excitement about biomedical research; raise aspirations, especially in the local community; widen participation in further and higher education; and to help improve health and wellbeing, especially in East London.

## Advantages to University Staff and Students

The presence of a science education and public engagement project within a working research laboratory has a number of positive impacts on QMUL’s biomedical and life sciences research and its researchers (summarised in Table 1). Stimulating a climate of enthusiasm for public engagement amongst staff, Centre of the Cell also allows researchers to effectively engage with the public to a high standard because they can combine their knowledge and expertise with that of professionals who have experience and training in public engagement with the life sciences. Centre of the Cell staff can also use their training to run lectures on science communication for undergraduate and postgraduate students.

**Table 1:** Advantages of siting a science centre in a research laboratory.

Engenders a climate of enthusiasm for public engagement with research
Improves science communication and public engagement skills of staff and students
Venue to aid recruitment of new students and staff
Allows high-quality and wide-ranging public engagement with research
Allows unique public engagement projects to be incorporated into research grant applications
Aids recrutiment of volunteers to population health research projects
Increases research impact

There are other benefits for undergraduates. A scheme funded by St Bartholomew’s Medical College Trust allows medical and dental students to work part time for Centre of the Cell. This gives Centre of the Cell a motivated, informed, and flexible work force and has also been of great benefit to the participating students, especially in enhancing their communication skills, as well as improving their finances. As many of the students live locally, they are excellent role models for Centre of the Cell’s younger visitors from East London. Postgraduate students, postdoctoral fellows, and more senior academics train as science, technology, engineering, and math (STEM) ambassadors and then volunteer to help with Pod shows, often giving impromptu talks about their own research. For instance, from 2013–2014 71% of all Pod shows from schools had a volunteer member of QMUL staff in attendance. Finally, Pod shows are a useful addition to university recruitment and open days and part of a campus tour for VIPs.

## Centre of the Cell Activities

Although Centre of the Cell’s activities cover a wide range of topics in biomedicine and the life sciences, they all relate to the project’s top-level message: *“Your body is made of millions of cells*. *People here and all around the world are trying to find ways to make cells better*. *You can help keep your cells healthy*.*”* This top-level message gives the project both identity and focus.

### The Pod Show

The Pod is at the heart of all Centre of the Cell activities. The design of the Blizard building allows visitors to see scientists at work while safely entering the Pod without disrupting the research environment (Fig 2). The major target audience is young people aged 8–18 and their families. Each Pod show, for 30–40 visitors, is an immersive theatrical experience lasting approximately 60 minutes that uses sound, lighting, film, digital interactive games, and objects to educate, inform, and engage with cell biology and biomedical research. Website content (e.g., digital interactive games and lesson plans) can be used pre- and postvisit to enhance the learning experience.

### Science Shows, Workshops and Lectures

**Fig 3:**
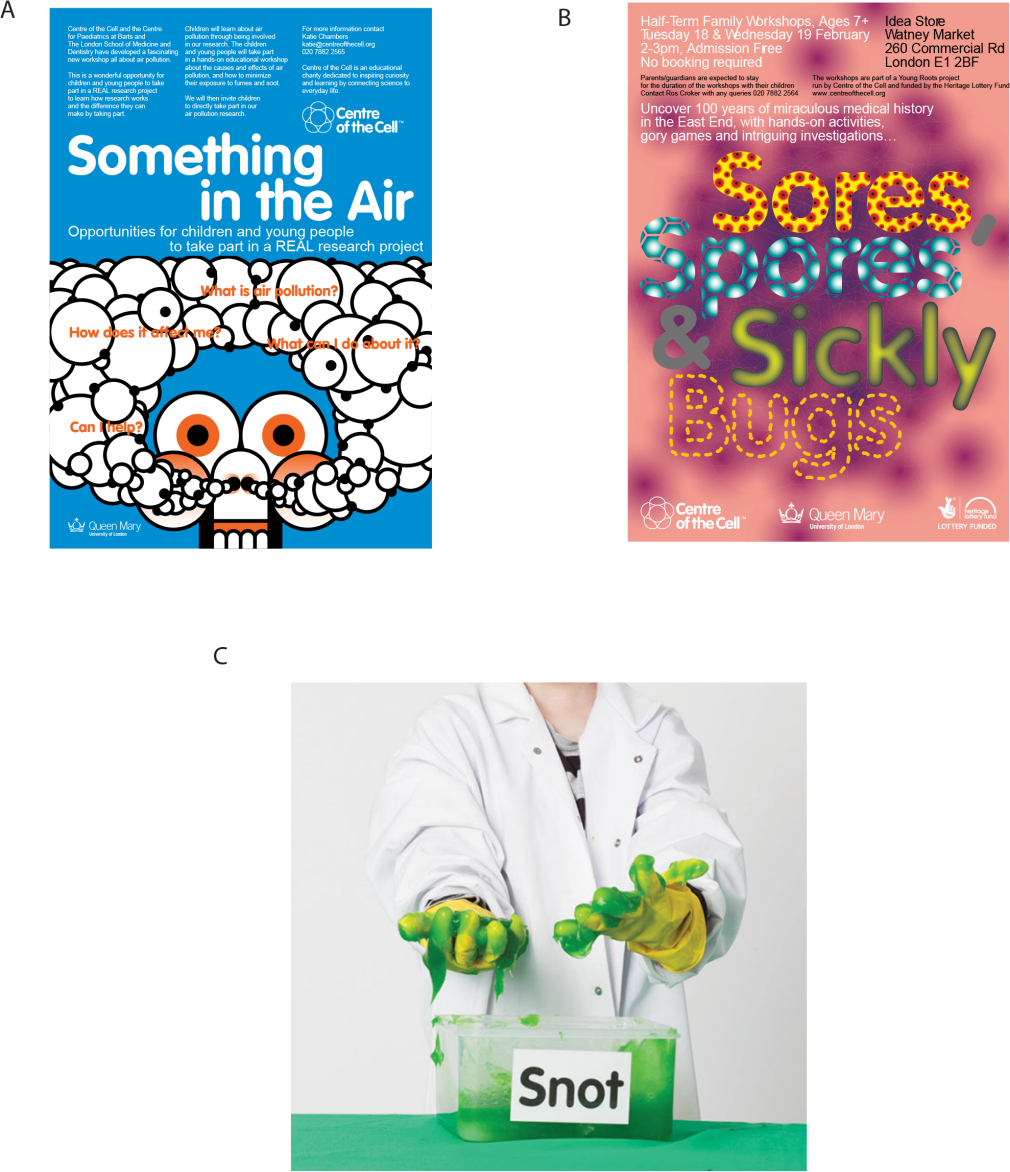
Centre of the Cell science shows. All shows and workshops have strong audience participation. **A)** and **B)** Posters advertising two of our science shows, target audience 8–12-year-olds. **C)** “Snot” (food dye preparation of methylcellulose) from immunology science show *Snot*, *Sick and Scabs*, target audience 8–12-year-olds. Images A and B are courtesy of Pony Ltd., London. Image C is courtesy of Centre of the Cell.

From the outset, the Pod shows were very popular with schools during term time and with family groups on school holidays, often booked at full capacity of three Pod shows each day, but it soon became apparent that the audiences wanted more. Hence, we have devised a number of science shows, workshops, and public lectures in collaborations between the local community groups, QMUL scientists, and the Learning Team at Centre of the Cell. There are now six science shows each lasting about 45 minutes with many opportunities for audience participation (with titles such as *Snot*, *Sick and Scab* and *Teethtastic*) and five workshops that also last for 45 minutes but have multiple activities or “stations” for the audience to engage with (for example, *Microbe Detectives*) (see Fig 3 for images from shows and workshops and Box 1 for more detail of one of the science shows). The topics for these shows and workshops were chosen both for their relevance to the research within the institution and for their links to the United Kingdom National Curriculum. Since 2011, twelve Big Question lectures have been delivered by leading experts for a general teenage audience with titles such as *Should you be allowed to genetically design your own baby*? and *Will there ever be a cure for cancer*?

**Box 1. Example of a Science Show—*Teethtastic***

*Teethtastic* was developed in partnership with the School of Dentistry in response to a demand from schools. *Teeth and bones* was a major component of the Key Stage 2 (upper primary) science curriculum of England and Wales at the time of development of this show. In addition, oral hygiene is a key concept in personal, health, and social education (PHSE) in primary schools in England and Wales.

The target audience for the show is Key Stage 2 (7–11-year-olds), although it is also popular with 11–13-year-old school groups and families visiting during school holidays. As well as covering the curriculum content for teeth and bones, the show aims to relate the content to the research work being conducted by scientists—in this case the use of the stem cells found in milk teeth in research.

The key messages are:

- What are teeth?
- What happens when we lose our milk teeth?
- Which cells can be found in teeth and what are their functions?
- How many teeth do humans have?
- The shape and function of the different types of human teeth
- Comparison of human teeth to those of other animals
- Lifestyle choices for healthy teeth—focusing on diet and oral hygiene

### Widening Participation and the Youth Membership Scheme

After two years of operation, local teenagers who were especially interested in medicine and life sciences began contacting the team at Centre of the Cell with enquiries about work experience and science careers. Hence, Centre of the Cell’s Youth Membership Scheme (YMS) began. Centre of the Cell is a teenager-friendly portal to the science world—young people often find out about us through school trips, and this seems to make us approachable.

The YMS is open to young people aged 14–19 and offers work experience, volunteering, and hospital placement opportunities, career advice, and the chance to meet scientists and healthcare professionals, as well as mentoring and revision sessions with scientists, medical, and dental students and Centre of the Cell staff. There is also a Youth Forum who are engaged in the front-evaluation of all new content ideas and have opportunities to take part in special projects. For instance, during 2013–2014, the Heritage Lottery Fund funded a group of 14–18-year-olds to research the East End’s medical history. Their aim was to understand and translate key changes in practice and knowledge into a science show that they would then share with family audiences at Centre of the Cell. The group committed to weekly workshops with additional school holiday sessions and museum visits over the course of a year, working with the Royal London Hospital Museum and Archives. With help from Centre of the Cell’s Learning Team, they developed the skills, knowledge, and understanding of how to design and lead sessions for family audiences, working as a team to create scripts, costumes, and props. The resulting show, *Spores*, *Sores and Sickly Bugs*, was a great hit with young people and their families, with positive feedback provided about the fun, interactive, and informative nature of the activities they created. The youth team then completed their project by training the next generation of Youth Members to deliver the show to future audiences.

**Fig 4:**
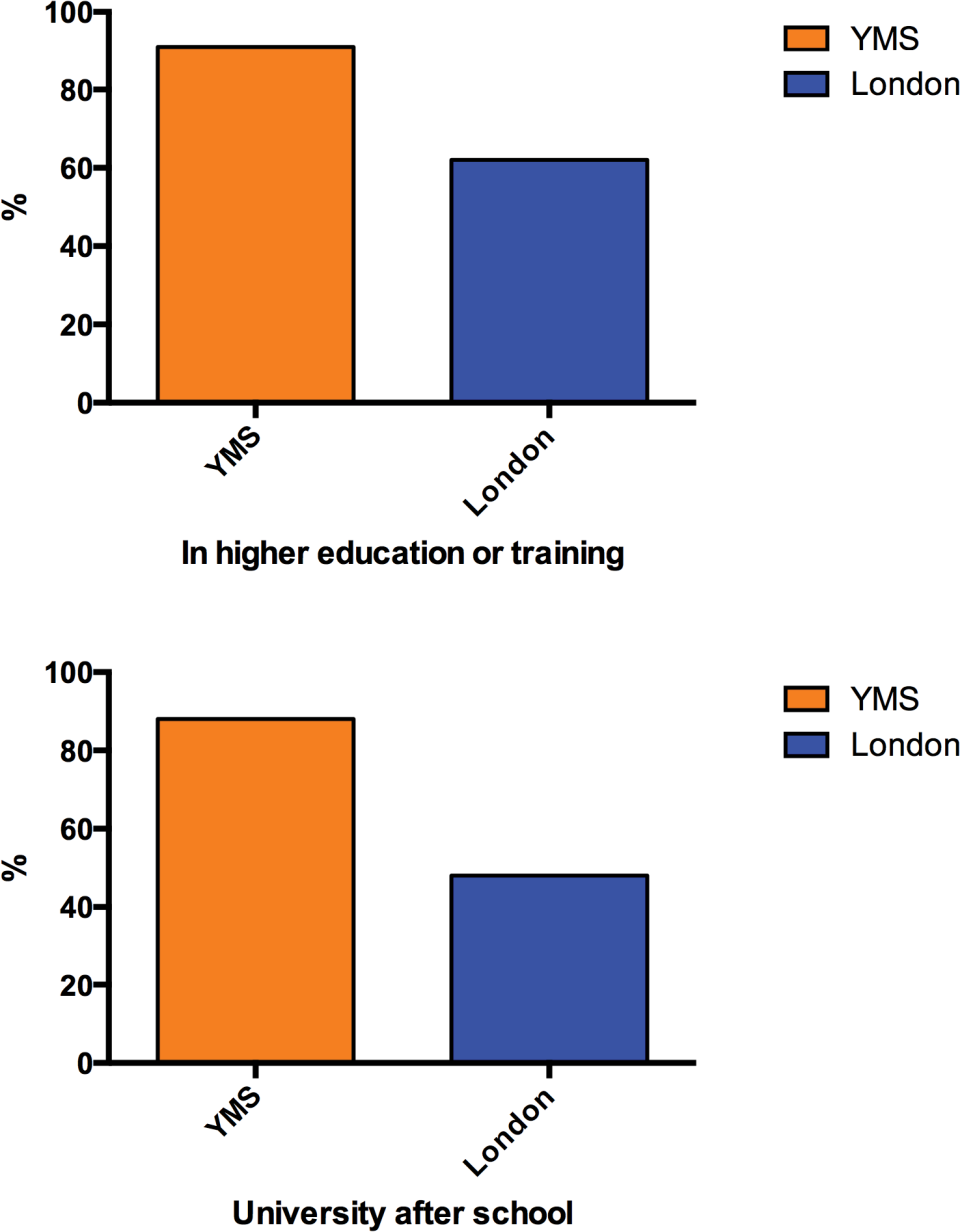
Higher education destinations of Centre of the Cell Youth Members 2015. Data obtained from 130 former Youth Members in comparison with London figures for Key Stage 5 school leavers (young people aged 17–19).

It is too soon to properly evaluate the impact of the YMS, but there are some encouraging initial data. Of 130 youth members who have now left school that we have been able to contact, 91% are in education or training compared to 62% of 2013 Key Stage 5 leavers in London (Fig 4A), 88% are at university compared to 48% in higher education of 2013 Key Stage 5 leavers in London (Fig 4B). http://www.london.gov.uk/sites/default/files/Education-Report-2014.pdf. 79% of these past YMS members are studying STEM subjects in higher education and 20% are studying at QMUL or its medical school. While collecting these data, staff received encouraging feedback from the young participants about the ways in which the YMS had influenced their career choices, had helped them with university applications, and provided them with relevant work experience opportunities. Box 2 contains two case histories of Youth Members who are now in higher education.

**Box 2. Two Case Histories of Centre of the Cell Youth Members**

Nobith started volunteering with Centre of the Cell in Summer 2009, immediately after completing his General Certificate of Secondary Educations (GCSEs). During his time volunteering, Nobith found the advice and support he received from the team invaluable—such as advice on university life, help with A level choices, and interview practice. This advice led to him to consider applying to universities outside London, such as Cambridge University. Nobith started studying medicine at Cambridge in 2011, with the generous support of an Aldgate and Allhallows Foundation scholarship, which was obtained with the assistance of Centre of the Cell staff. In 2014, he moved to Barts and The London to complete his clinical years, and he plans to start volunteering with Centre of the Cell again in Summer 2015. As Nobith said in a recent meeting with us:

*“Without Centre of the Cell, I wouldn’t have applied for Cambridge.”*

*“Centre of the Cell has been there for me—I won’t forget how they have supported me throughout.”*

Jhanara joined the YMS when she was in Year 10 (age 14–15). After volunteering in Centre of the Cell family events, she took part in the 2013–2014 “Young Roots” project, which brought together a team of young people aged 14–18 from across East London to investigate the medical heritage of the East End. The team created webpages and a family workshop, *Sores*, *Spores and Sickly Bugs*, which they presented to the public. Jhanara started studying Biomedical Science at University of Greenwich in 2014. Jhanara says, *“Centre of the Cell has helped me greatly*. *I was able to develop many skills such as public speaking*, *teamwork*, *planning and organisation*, *and also problem solving*. *I was able to use this experience in my UCAS (university) application*.*”*

### Evaluation and Impact

Between 2003 and 2009, Centre of the Cell conducted a comprehensive front-end evaluation of the ideas, learning aims, and top-level message of the project with 9,000 school pupils, teachers, and members of the public from local East London schools and community groups. This evaluation assessed potential target audiences’ needs and interests, what they expected to see and do in the Centre, and their knowledge and interest in cell biology and biomedicine. When asked “what is a cell?” the replies ranged from “prison cell” and “cell phone” to “battery cell.” We also assessed the needs of visitors with disabilities and conducted subject-specific evaluation to determine target audiences’ knowledge and interest of particular subjects, including diseases, medicine, microscopes, and the acceptability of displaying specimens of human organs (the latter evaluation was extremely popular as we took human specimens from our pathology museum into school classes). Not only did this have a strong impact on the evolution of the project but also it engendered a feeling of “ownership” in our community and built up a postopening audience. Between 2010 and 2013, 81% of primary schools in the local borough of Tower Hamlets and 100% of the secondary schools had participated in Centre of the Cell activities.

The website and digital interactive games were also evaluated pre- and during development through the use of prototypes and feedback collected in small focus groups. This feedback was vital in determining whether the resources were user-friendly, enjoyable, and that they achieved the learning objectives.

Postlaunch, we have used feedback forms to assess whether the key goal of inspiring and motivating the project participants has been achieved; the effect on career choices and educational aspirations and what we have learned and how we can enhance the learning, mentoring, and training experience. The overall response is positive, especially in terms of achieving learning outcomes and accessing higher education. From the evaluation data to date, 76% of the young people stated that they “enjoyed” or “really enjoyed” their session. 75% were more interested in science after the session, and 71% said they felt they knew more about university.

### Development of New Public Engagement Activities

Continued success is dependent on development of new activities and content. Being part of a research university means there is a limitless and ever-changing resource of new science stories and scientific expertise. The original idea for a new activity can come from our scientists and clinicians and/or our community. As our research is so closely interwoven with the needs of the local East London community, Centre of the Cell can also facilitate research projects, especially in terms of recruitment of local volunteers.

Funding for new activities may be embedded in research grant applications—many funding bodies have a public engagement element and QMUL scientists now routinely incorporate funding for Centre of the Cell within their applications. These components may finance workshop or show development and delivery; digital interactive games that can be part of a Pod show and/or available online and/or as a free standing app; a Big Question Lecture; or new website content. Funding for new activities can also come from outside sources such as trusts and foundations or from specific grants for public engagement projects.

Once funding is secured, we develop the activity according to a standard operating procedure that begins with definition of the objectives of the project, the desired learning outcomes and front-end evaluation of the activity with the target audience. The iterative development then moves through further defined stages in a three-way collaboration between Centre of the Cell staff, scientists, and target audiences.

One example of our recent work is a Medical Research Council-funded project on the impact of air pollution on London’s children. Working with a team of paediatricians lead by Professor Jonathan Grigg, Centre of the Cell devised a three-hour workshop, *Something in the Air*. The learning aims are 4-fold: What is air pollution? How does air pollution affect people? How can we measure air pollution? What can I do to avoid air pollution? The event, which is delivered in schools, also has six workstations where young people can explore the science behind the research. However, this is more than just a public engagement or science communication exercise. Those children whose parents have given informed consent are recruited into the research study during the workshop. The paediatrics team obtain sputum, urine, and DNA samples from them and conduct lung function and skin-prick allergy tests. Once recruitment is complete in Spring/Summer 2015, the team will return to the schools to update them on the findings of the research and evaluate the impact of the project on their understanding of air pollution and the work of scientists.

### Sustainability

During the past five years, Centre of the Cell has received major financial support from the host institution, with specific projects funded by a wide range of charitable trusts and foundations. Now that much of the new content is funded by a proportion of research grants obtained by our scientists, the aim is to achieve financial security by a combination of earned income from shows and workshops, as well as sponsorship for activities such as the YMS, with a decreasing contribution from the medical school and QMUL. Workshop and science show charging has been in place since 2012, and we began to charge for Pod shows in September 2013.

### What Have We Learnt So Far?

A science education centre inside a laboratory building can be inspirational both for visitors and research staff. To anyone considering a similar project, the key recommendations are to conduct detailed “front-end” evaluation with target audiences before the project begins, exploit fully the unique resource of the science stories being generated in the laboratory that houses the science centre, think about sustainability from the start of the project—and remember to provide adequate facilities for visitors (toilets, lunch areas, etc.). Apart from that, although it is certainly hard work and can be very challenging, our experience is that it is very rewarding and—importantly—great fun.

## Future Plans

There are three main areas for development in the near future. First, the project must reflect the fast moving pace of life sciences and biomedical research with new content and activities added on an annual basis and existing content refreshed. Keeping our digital Pod at the cutting edge of information technology (IT), we aim to fundraise for more display objects that we will bring to life with augmented reality technology.

**Fig 5:**
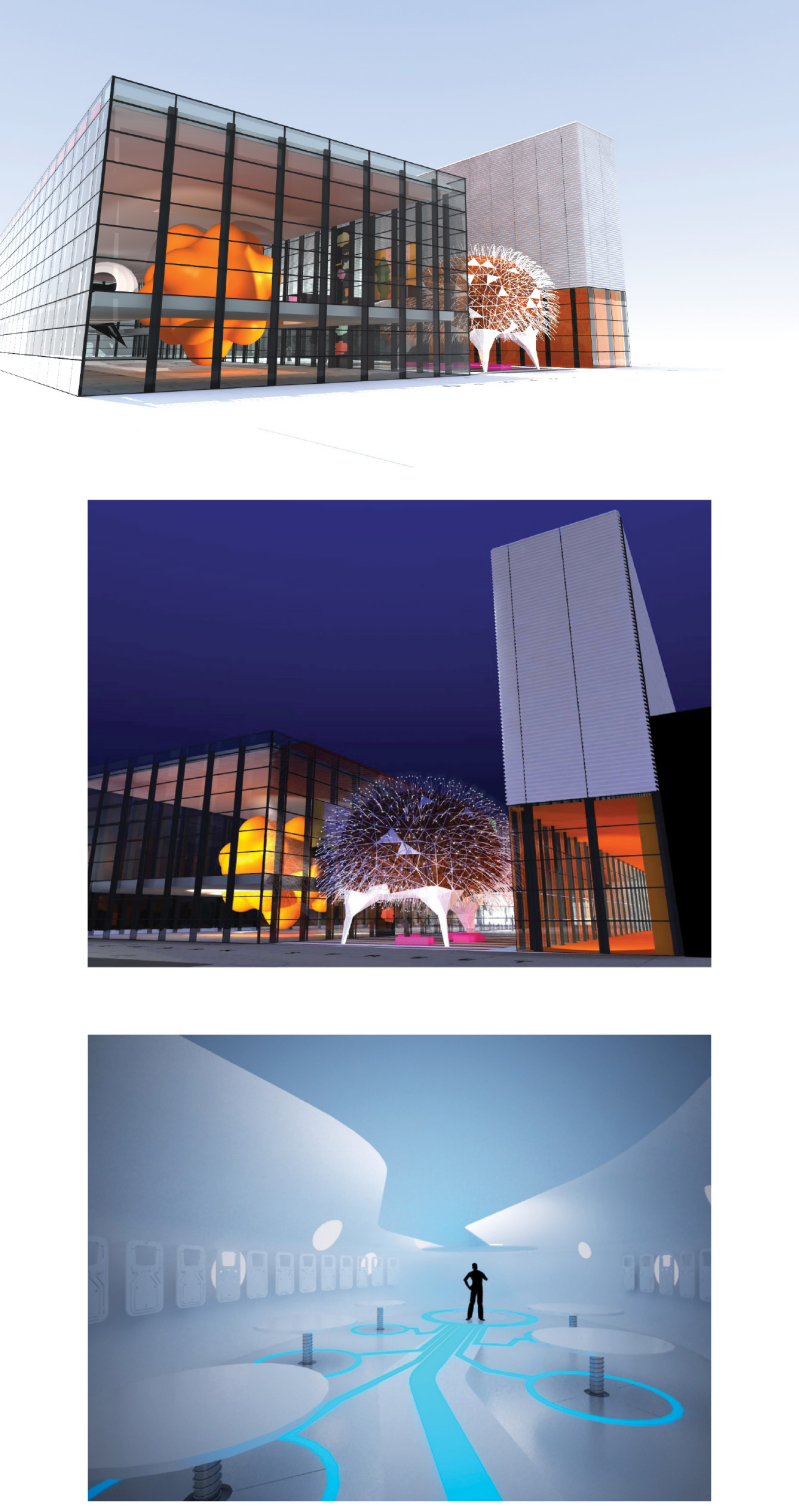
Artists’ impressions of Neuron Pod. Top ‒ Neuron Pod and its relationship to the orange Centre of the Cell Pod. Visitors will enter Neuron Pod via the same multicoloured bridge that leads to the other Pod. Centre ‒ Neuron Pod at night with fibre-optic “dendrites” on full display. Bottom ‒ The interior of Neuron Pod—a neutral, multipurpose, and reflective space with tables that rise up from the floor when needed. Top and centre images courtesy of ALL Design. Bottom image courtesy of Land Design Studio.

The second priority is project sustainability. This will most likely be achieved by adequate but competitive charging for all activities combined with targeted funding applications for specific projects. A third priority, which will have major impact, is the successful completion of a capital fundraising campaign for a second Pod, Neuron Pod (see Fig 5), to be positioned in the Blizard Mews and accessed by the same bridge as the existing Pod. This has potential to double visitor numbers and dwell time on site, providing a dedicated space for all science shows, workshops, and youth member activities, as well as providing opportunities for new adult initiatives and at the weekend.

## Abbreviations

GCSE: General Certificate of Secondary Education
IT: information technology
PHSE: personal, health, and social education
QMUL: Queen Mary University of London
STEM: science, technology, engineering, and math
YMS: youth membership scheme

